# Increasing access to psychological services within pediatric rheumatology care

**DOI:** 10.1186/s12969-023-00837-4

**Published:** 2023-06-02

**Authors:** Alana Goldstein-Leever, Christine Bearer, Vidya Sivaraman, Shoghik Akoghlanian, James Gallup, Stacy Ardoin

**Affiliations:** 1 Nationwide Children’s Hospital, Columbus, OH USA; 2 The Ohio State University, Columbus, OH USA; 3 Department of Psychology, Nationwide Children’s Hospital, 700 Children’s Drive, J West 3rd Floor, Columbus, OH 43205 USA; 4 Akron Children’s Hospital, Akron, OH USA; 5 Center for Clinical Excellence, Nationwide Children’s Hospital, Columbus, OH USA

**Keywords:** Mental health, Quality improvement, Pediatric rheumatology

## Abstract

**Background:**

Given the impact of psychological factors on rheumatic disease, pediatric psychologists serve a vital role in promoting quality of life and managing common problems among youth with rheumatic disease. The aim of this project was to increase access to psychological services among youth with rheumatic disease at a children’s hospital.

**Methods:**

A quality improvement (QI) team identified key drivers and interventions aimed to increase access to psychological services for youth with rheumatic disease. Data was collected for a 6-month baseline period and 4-year intervention period. We applied the Plan-Do-Study Act method of QI and the American Society for Quality criteria to adjust the center line and control limits.

**Results:**

There were two statistically significant center line shifts in the number of patients seen by psychology and one statistically significant shift in referrals to psychology over time with applied stepwise interventions. Patients seen by a psychologist increased by 3,173% from a baseline average of 1.8 to 59.9 patients seen per month (*p* < 0.03). Psychology referrals increased by 48% from a baseline average of 9.85 to 14.58 referrals per month over the intervention period (*p* < .01).

**Conclusions:**

Youth with rheumatic disease received increased access to mental health treatment when psychological services were imbedded within rheumatology care. Psychology referrals also increased significantly, suggesting that psychology integration within a medical clinic can increase identification of needs. Results suggest that psychology integration into rheumatology care may increase access to mental health treatment and identification of psychological needs in this at-risk population.

There is a high prevalence of emotional disorders, including depression and anxiety, among youth with rheumatic disease [1]. International estimates of depression and anxiety among patients with juvenile idiopathic arthritis (JIA), juvenile systemic lupus erythymus (jSLE), and fibromyalgia range from 15 to 65% [2]. Symptoms of depression and anxiety among youth with rheumatic disease are associated with increased symptom expression, functional limitations, and healthcare utilization, as well as poorer medication adherence and health-related quality of life [1, 3–5]. Likewise, the demands of chronic illness, pain, and longstanding medical treatment may increase the likelihood of emotional distress and disorders [1]. The impact of the COVID-19 pandemic on children and adolescents also remains profound, exacerbating mental health needs and increasing the demand for psychological services [6]. It remains important to note that early recognition and treatment of mental health disorders improves emotional and physical health outcomes for children and adolescents [7]. However, gaps in access to mental health care have been well-documented for youth with rheumatic disease [8].

The Childhood Arthritis & Rheumatology Research Alliance (CARRA) has issued guidance statements on the screening and management of mental health symptoms in pediatric rheumatology clinics, which support the relevance of mental health disorders within pediatric rheumatology and suggest the need for mental health problems to be treated as an aspect of pediatric rheumatology care [9]. Increasing mental health awareness and knowledge among rheumatologists remains paramount to identify mental health needs in youth with rheumatic disease. Psychologists and other behavioral health clinicians may serve as a valuable resource in the education of rheumatologists and direct provision of mental health care, given that rheumatologists are tasked with substantial competing demands in their management of medical care and rheumatic disease. Of note, psychological services have been successfully integrated into primary care and other specialty care settings, such as pediatric endocrinology and oncology, with demonstrated positive outcomes [10, 11].

Pediatric psychologists are trained in the areas of child and health psychology, with unique expertise in the screening of psychiatric disorders, management of acute and chronic illness, and health promotion [12]. Given the impact of psychosocial and behavioral factors on rheumatic disease, pediatric psychologists may serve a vital role in promoting quality of life and managing common presenting problems among youth with rheumatic disease, including adjustment to chronic illness and their treatment regimen, management of pain and emotional disorders, as well as promotion of healthy lifestyle behaviors [13]. Integration of psychological services into routine medical care offers patients and families the opportunity for increased access to evidence-based psychological assessment and treatment services, while limiting barriers to engagement and treatment, such as mental health stigma and lengthy wait times for treatment. To date, the authors are not aware of any other pediatric rheumatology practices that have fully integrated psychological services into routine rheumatology care. The aim of this project was to integrate psychological services into pediatric rheumatology care and increase access to psychological services among youth with rheumatic disease at a children’s hospital, as measured by psychology contacts and referrals to psychology. As a secondary outcome measure, the authors examined annual changes in depression scores among youth with jSLE who received psychological treatment as a standard of their routine medical care.

## Methods

A QI team was assembled and consisted of a psychologist, rheumatologists, a rheumatology fellow, and quality improvement specialist. This QI team identified key drivers and interventions aimed to increase access to psychological services for youth with rheumatic disease. The Plan-Do-Study Act method of quality improvement was applied. Authors followed the Standards for Quality Improvement Reporting Excellence (SQUIRE 2.0) Guidelines in the creation of this manuscript. Authors received ethics board approval and exemption from the institutional review board (IRB # STUDY00002401). Written informed consent was waived based on the IRB determination that this project is not research involving human subjects.

Data was collected for a 6-month baseline period (April through September 2017) and 4-year intervention period (October 2017 through November 2021). Data from baseline and intervention periods were obtained by extraction from the electronic health record. A psychology charge within an encounter was used as a proxy for psychology contact with a patient. Referrals to psychology were provided by rheumatology physicians and fellows, or the rheumatology psychologist. Referrals addressed common presenting problems that affect youth with rheumatic disease, including adjustment to chronic illness and treatment demands, medical adherence, and needle phobia, as well as management of acute and/or chronic pain, anxiety, and depression, and promotion of healthy lifestyle behaviors. Psychological services specifically targeted presenting problems that affect the patient; however, if caregiver or sibling needs, and/or family dynamics were directly impacting the patient, the psychologist provided either direct intervention or recommendations to address these family concerns. Psychology referrals were routinely reviewed by an intake coordinator and rheumatology psychologist to determine their appropriateness. Evidence-based screening tools, such as the PHQ-9, were used to inform referral decisions along with patient and family interview and provider observations.

**Fig. 1:**
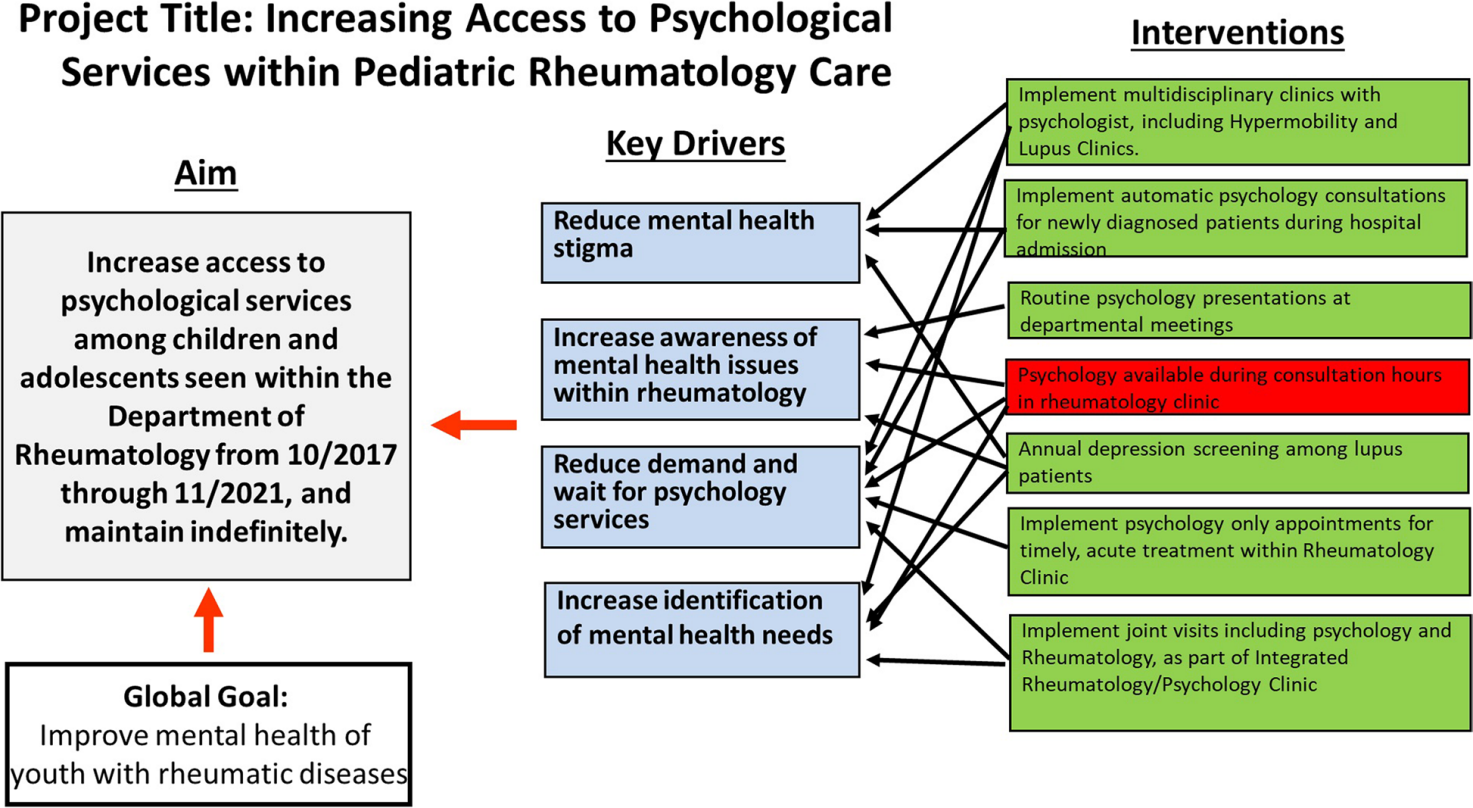
Key driver diagram

Key drivers for this project were identified as reducing mental health stigma, increasing awareness of mental health issues within pediatric rheumatology, reducing the demand and waitlist for psychological services, and increasing identification of mental health needs. See Fig. 1 for key driver diagram.

Interventions were performed over a 4-year period to increase access to psychological services among youth with rheumatic disease and to promote integration of psychological services into pediatric rheumatology care at a children’s hospital. The first intervention included a staffing change (i.e., the existing psychologist supporting the rheumatology service was replaced by a new psychologist) and implementation of a multidisciplinary clinic for patients with jSLE, which included psychology. As part of this multidisciplinary clinic, patients with jSLE were offered psychological services in addition to their rheumatology and nephrology care, and support from social work and pharmacy services. The second intervention involved initiation of annual depression screening with the Patient Health Questionnaire-9 (PHQ-9) to patients with jSLE as a standard of care. The third intervention included implementation of a multidisciplinary clinic for patients with joint hypermobility and Ehlers-Danlos syndrome. As part of this intervention, psychology closely followed these patients and intervened to support management of chronic pain, anxiety and depression, as well as other behavioral challenges. Youth with jSLE and hypermobility were selected as the first patient groups to receive integrated psychological services based on the demonstrated behavioral needs within these subpopulations, as well as the authors’ desire to implement quality improvement interventions on a smaller sample to ensure feasibility and troubleshoot any challenges associated with our process. For the fourth intervention, an integrated clinic was created in order to provide youth with any diagnosis of rheumatic disease with access to joint psychology and rheumatology appointments. For the fifth and final intervention, psychology only appointments were made available within rheumatology clinic in order to allow timely access to psychological services within the medical clinic setting and reduce barriers associated with seeking outpatient psychological services in a behavioral health clinic.

Statistical process control was employed to monitor data throughout the course of this quality improvement project. We applied the American Society for Quality criteria to adjust the center line and control limits, evaluated each of our outcomes monthly, and modified interventions as needed. Individual measurements control charts (I-Charts) [14] were performed to track improvement over time and t-tests were performed to assess for statistical significance. A run chart was performed to track the percentage of patients with jSLE who demonstrated a decreased or stable score on a standardized measure of depression (the PHQ-9) over time.

## Results

**Fig. 2:**
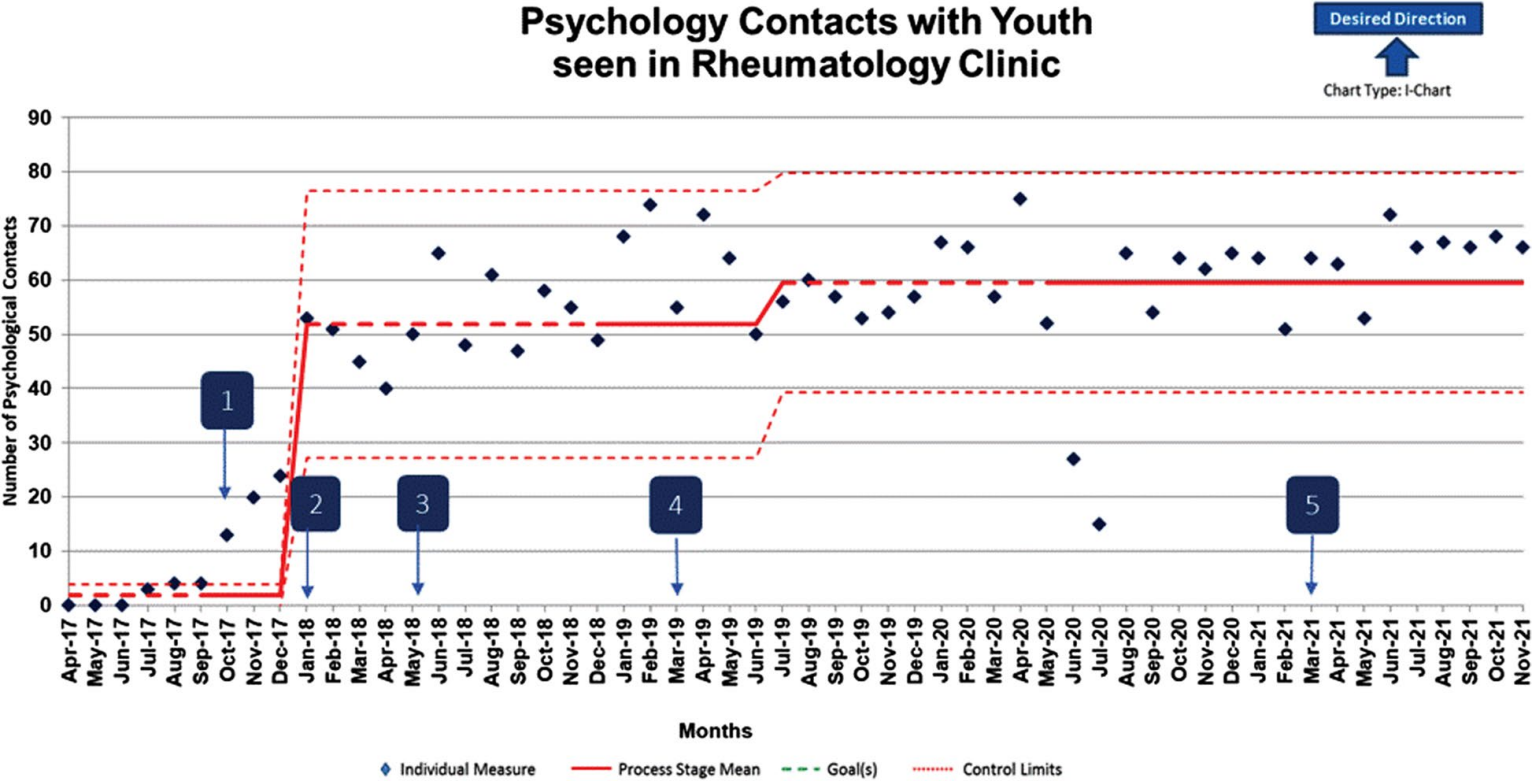
I-Chart indicates number of youth with rheumatic disease seen by psychologist over time. Annotation Legend: 1. October 2017: Initiate multidisciplinary lupus clinic; New psychologist replaces former psychologist. 2. January 2018: Implement depression screening in jSLE. 3. May 2018: Initiate multidisciplinary hypermobility clinic. 4. March 2019: Implement integrated psychology/rheumatology clinic. 5. March 2021: Implement psychology only visit type within rheumatology clinic

The sample was primarily female and Caucasian, with an average age of 13. See Table 1 for demographic characteristics of the current sample. The I-chart featured in Fig. 2 highlights the number of patients with rheumatic disease who were seen by a psychologist over the intervention period. There were two statistically significant center line shift in the number of patients seen by psychology over time with applied stepwise interventions. Specifically, patients seen by a psychologist increased by 3,173% from a baseline average of 1.8 to 59.9 patients seen per month from April 2017 to November 2021 (*p* < 0.03).

**Table 1:** Demographic characteristics of youth with rheumatic disease seen by psychology from October 2017 through November 2021 (*N* = 760)

	**M (SD)**
Age	13 ( 4.3)
	**N (%)**
Assigned Sex^a^	
Male	195 (25.7)
Female	565 (74.3)
Race/Ethnicity^b^	
White/Caucasian	594 (78.5)
African American	86 (11.4)
Biracial or Multiracial	38 (5)
Latino/Hispanic	23 (3)
Asian	12 (1.6)
Native Hawaiian and Pacific Islander	4 (0.5)

Legend. *M* Mean, *SD* Standard deviation. Parentheses indicate percentages.

^a^ Data on gender identity was unavailable

^b^ Includes missing data, in which, caregivers preferred not to respond

**Fig. 3:**
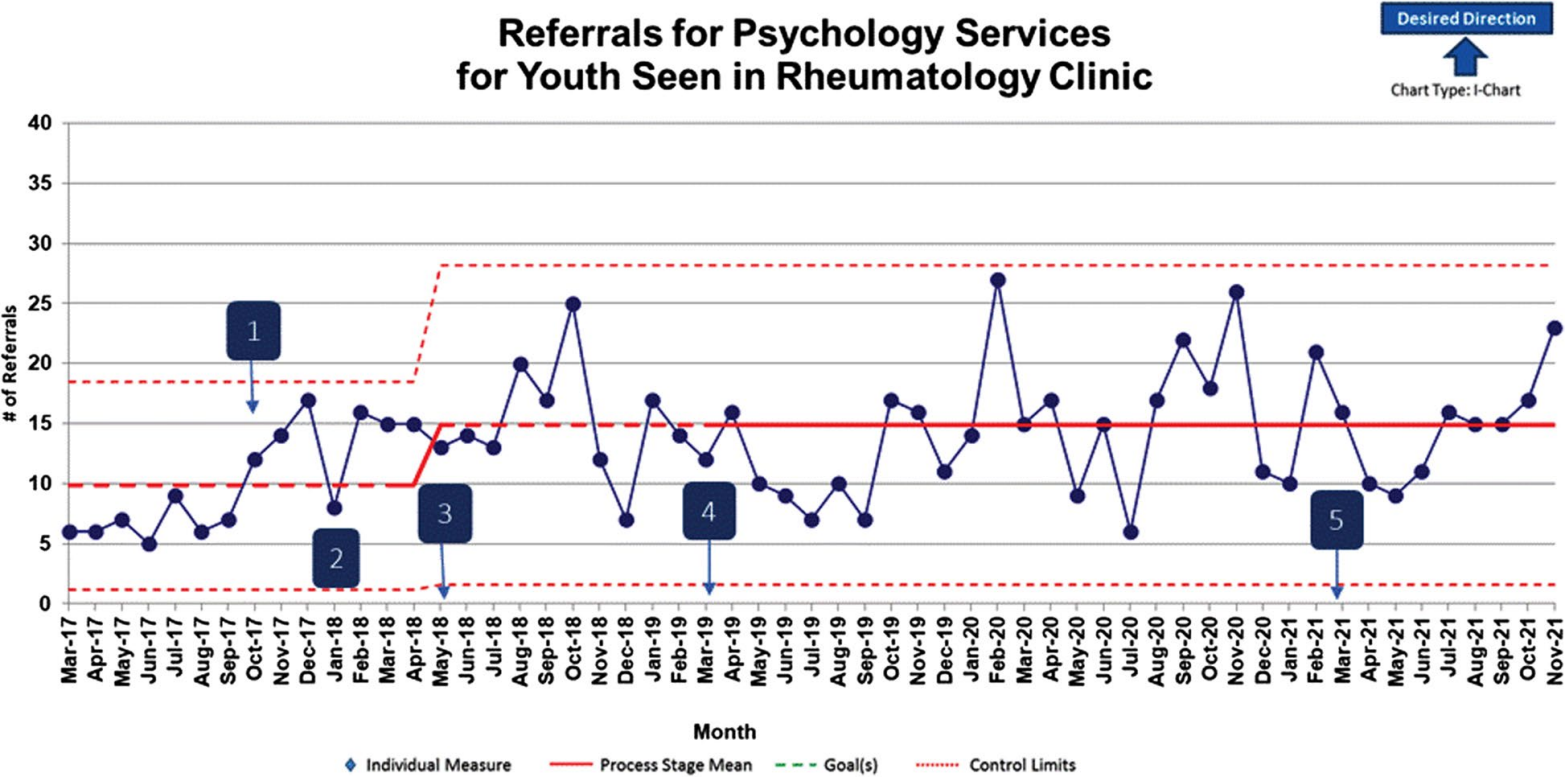
I-Chart indicates number of psychology referrals made by rheumatology providers and rheumatology psychologist. Annotation Legend: 1. October 2017: Initiate multidisciplinary lupus clinic; New psychologist replaces former psychologist. 2. January 2018: Implement depression screening in jSLE. 3. May 2018: Initiate multidisciplinary hypermobility clinic. 4. March 2019: Implement integrated psychology/rheumatology clinic. 5. March 2021: Implement psychology only visit type within rheumatology clinic

**Fig. 4:**
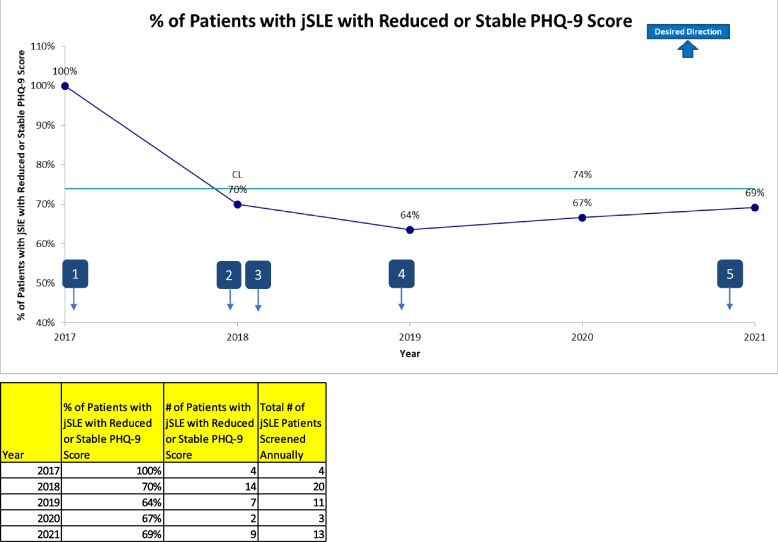
Run chart depicts annual changes in depression scores among youth with jSLE who received psychological treatment as a standard of their routine medical care. Annotation Legend: 1. October 2017: Initiate multidisciplinary lupus clinic; New psychologist replaces former psychologist. 2. January 2018: Implement depression screening in jSLE. 3. May 2018: Initiate multidisciplinary hypermobility clinic. 4. March 2019: Implement integrated psychology/rheumatology clinic. 5. March 2021: Implement psychology only visit type within rheumatology clinic

Figure 3 depicts the number of referrals to psychology for patients with rheumatic disease being followed for outpatient rheumatology care. There was a statistically significant center line shift in the number of patients referred to psychology over time. Specifically, psychology referrals increased by 48% from a baseline average of 9.85 to 14.58 referrals per month over the intervention period from March 2017 to November 2021 (*p* < 0.01). Common reasons for psychology referral within this patient population included: needle fears and phobia, pain management, and treatment of anxiety and depression. Seasonal shifts in referral frequency were observed in line with seasonal variations in stress level and school demands for patients and families, as well as pandemic conditions. Additionally, Fig. 4 demonstrates a trend, in which, the majority of patients with jSLE who received psychological services during rheumatology care maintained a reduced or stable PHQ-9 score across annual screenings for depression.

Patient satisfaction data was collected as part of the current study, which revealed an average rating of 4.6 on a 0–5 scale. Qualitative review of responses showed that patients and families perceived a benefit from receipt of psychological services within the rheumatology clinic setting. Patients and families specifically indicated feeling comfortable meeting with the psychologist, having increased access to mental health treatment as a function of our programming, and that psychology involvement helped them to better manage their rheumatic diseases. Furthermore, patients and families reflected upon their increased comfort level upon receiving psychological care within their rheumatology clinic and expressed appreciation for being able to forgo a separate behavioral health or psychological evaluation outside of the medical setting. Based on qualitative feedback from the physician team, psychology involvement in rheumatology care allowed physicians to spend reduced time focusing on the behavioral factors that impact disease management and thereby, increased provider efficiency.

## Discussion

Youth with rheumatic disease are at increased risk for mental health disorders, though availability of mental health care remains limited in this population. We found that youth with rheumatic disease received increased access to mental health treatment when psychological services were integrated and embedded within their routine rheumatology care. Referrals to psychology also increased significantly over the intervention period, suggesting that psychology integration within a medical clinic can increase identification of psychosocial and behavioral needs among patients and families. Additionally, the majority of youth with jSLE demonstrated reduced or stable depression scores over time when receiving psychological treatment as a component of their medical care. Results of this project suggest that psychology integration into rheumatology care remains feasible, and increases access to mental health treatment and identification of psychological needs in this at-risk population. Of note, there were no established models to follow for integration of psychological services into pediatric rheumatology care at the time of this project’s implementation. This integrated model represents the first of its kind to include psychology service embedment throughout all aspects of pediatric rheumatology care.

Extant literature supports the merging medical and psychology specialties into an integrated plan of care to improve mental and physical health outcomes in youth with chronic illness [15]. In line with a biopsychosocial approach to care, we have found substantial value added by integration of psychological services into routine rheumatology care. Psychology integration into medical care has been shown to reduce mental health stigma and barriers to care [15]. This was observed within our population where access to psychological services proved invaluable in normalizing discussion of mental health concerns, and promoting patient and family’s buy-in and follow through with mental health evaluation and treatment. It remains important to note that our patient population represented a large catchment area, including a significant percentage of patients from rural areas and two neighboring states with poor access to mental health services. As such, embedment of psychological treatment into medical care allowed for increased access to behavioral health care. Patient satisfaction data revealed an overwhelmingly positive response to our integrated model, with patients and their families expressing satisfaction and appreciation of psychology’s integration into rheumatology clinic, and reflecting upon their increased access to mental health treatment and improved disease management.

This project was completed over the course of 4 years, in which psychology was gradually integrated into pediatric rheumatology care. Our QI team has learned the value of approaching integration with a stepwise progression to ensure that team members are aware of mental health needs among youth with rheumatic disease, as well as the contribution that psychological services lend to a patient’s plan of care. Psychological services were first provided to youth with rheumatic disease within a separate behavioral health clinic and targeted common reasons for referral, such as anxiety/needle fears, pain management, and depression. Clinical screening was later introduced to identify patients with mental health or behavioral needs who were then seen by psychology across behavioral health clinic or multidisciplinary clinic settings. Psychological services were strategically integrated into multidisciplinary clinics within rheumatology, particularly among populations with high needs (e.g., those with jSLE or joint hypermobility), and eventually, expanded to patients with wide-ranging diagnoses within the rheumatology clinic setting. Primary interventions within this quality improvement initiative included implementation of mental health-focused education among providers and staff, mental health screening, and multidisciplinary medical clinics with psychology involvement. Psychological services were made available on a preventative basis for those with a new diagnosis or within certain high-risk groups, as well as when patients were presenting with acute problems that were in need of treatment.

There are barriers to psychology integration into rheumatology care, including limited access to or funding for a psychologist or mental health professional. Should resources be available to obtain a psychologist, other barriers may include team cohesion and awareness of mental health impact on physical functioning and psychology’s role, as well as the psychologist’s ability to remain productive with a sufficient referral base. There were also time demands associated with psychology integration into rheumatology care. Integrating psychological services into rheumatology care increased visit duration by approximately 30 to 60 min, though patients and families consented to extend their visit, and clinical flow and templates were adjusted accordingly. Patients were scheduled within a lengthier time slot when behavioral needs were anticipated to ensure sufficient time for evaluation and treatment of rheumatic disease, as well as provision of psychological services. Alternatively, when behavioral needs were discovered spontaneously during the visit, psychological intervention directly followed medical management to limit disruptions to the clinic flow and the physician’s schedule. Based on qualitative feedback, psychology’s involvement allowed providers to spend reduced time focusing on the behavioral factors that impact disease management and thereby, increased provider efficiency.

Future steps within our program include broadening of our mental health screening to include assessment of anxiety and suicide risk, ensuring access to screening across rheumatic diseases, as well as increasing the scope of psychology integration into rheumatology clinic and outcome measurement in the form of provider satisfaction with integrated services. Additionally, future studies of interest include exploration of the impact of psychology referral and involvement on rheumatic disease scales, such as patient global score.

Psychology integration into pediatric rheumatology care allowed for exponential growth in access to mental health support, at a time where children and adolescents are experiencing heightened levels of emotional distress, especially those facing additional risk factors of chronic physical illness and pain. Through integration of psychological services into rheumatology care, receipt of mental health treatment was normalized and de-stigmatized within our patient population. Psychosocial and geographical barriers for patients were also minimized by embedding psychological services into established medical care. Physicians also gained an appreciation for the contribution of psychological services in their practice upon observing improved outcomes for their patients when psychology was involved, as well as the reduction in the demands on rheumatologists when behavioral targets were instead addressed by a psychologist. This study suggests the feasibility of psychology integration into pediatric rheumatology care, and highlights the capacity for psychology integration to increase access to mental health and behavioral support among patients and families affected by pediatric rheumatic disease.

## Conclusions

Youth with rheumatic disease received increased access to mental health treatment when psychological services were integrated and embedded within pediatric rheumatology care. Referrals to psychology also increased significantly over the intervention period, suggesting that psychology integration within a rheumatology clinic can increase identification of psychosocial and behavioral needs among youth and families. Results of this project suggest that psychology integration into rheumatology care remains feasible, and increases access to mental health treatment and identification of psychological needs in this at-risk population. While psychological services have been increasingly integrated into pediatric primary care and other specialty care settings [10, 11], no current models or research exist for the integration of psychological services into pediatric rheumatology. This manuscript describes an integrated model of pediatric rheumatology care and highlights novel interventions that may be employed to embed psychological support within a rheumatology clinic and increase access to psychological services for youth with pediatric rheumatic disease.

## Data Availability

The datasets used and/or analyzed during the current study are available from the corresponding author on reasonable request.

## Abbreviations

JIA: Juvenile idiopathic arthritis
jSLE: Juvenile systemic lupus erythematosus
QI: Quality improvement
(CARRA: Childhood Arthritis & Rheumatology Research Alliance
SQUIRE 2.0: Standards for Quality Improvement Reporting Excellence
IRB: Institutional review board
PHQ-9: Patient Health Questionnaire-9
I-Chart: Individual measurements control chart
